# Meta‐analysis using individual participant data: one‐stage and two‐stage approaches, and why they may differ

**DOI:** 10.1002/sim.7141

**Published:** 2016-10-16

**Authors:** Danielle L. Burke, Joie Ensor, Richard D. Riley

**Affiliations:** ^1^ Research Institute for Primary Care and Health SciencesKeele UniversityStaffordshireU.K.

**Keywords:** individual patient data, individual participant data, meta‐analysis, IPD, one‐stage, two‐stage

## Abstract

Meta‐analysis using individual participant data (IPD) obtains and synthesises the raw, participant‐level data from a set of relevant studies. The IPD approach is becoming an increasingly popular tool as an alternative to traditional aggregate data meta‐analysis, especially as it avoids reliance on published results and provides an opportunity to investigate individual‐level interactions, such as treatment‐effect modifiers. There are two statistical approaches for conducting an IPD meta‐analysis: one‐stage and two‐stage. The one‐stage approach analyses the IPD from all studies simultaneously, for example, in a hierarchical regression model with random effects. The two‐stage approach derives aggregate data (such as effect estimates) in each study separately and then combines these in a traditional meta‐analysis model. There have been numerous comparisons of the one‐stage and two‐stage approaches via theoretical consideration, simulation and empirical examples, yet there remains confusion regarding when each approach should be adopted, and indeed why they may differ.

In this tutorial paper, we outline the key statistical methods for one‐stage and two‐stage IPD meta‐analyses, and provide 10 key reasons why they may produce different summary results. We explain that most differences arise because of different modelling assumptions, rather than the choice of one‐stage or two‐stage itself. We illustrate the concepts with recently published IPD meta‐analyses, summarise key statistical software and provide recommendations for future IPD meta‐analyses. © 2016 The Authors. *Statistics in Medicine* published by John Wiley & Sons Ltd.

## 1 Introduction

Statistical methods for meta‐analysis and evidence synthesis are increasingly popular tools in medical research, as they synthesise quantitative information across multiple studies to produce evidence‐based results. An aggregate data meta‐analysis is the most common approach, where summary study results (such as treatment effect estimates and their standard errors) are obtained from study publications or study authors, and then synthesised. This approach is, at least in principle, relatively quick and inexpensive, but it often faces problems such as poor and selective reporting in primary studies, publication bias and low power to detect individual‐level interactions such as how a participant‐level covariate modifies treatment effect 1, 2. An individual participant data (IPD) meta‐analysis can help overcome many of these issues, by obtaining and then synthesising the raw, participant‐level data from each study. For example, IPD allows the meta‐analyst to standardise the inclusion criteria and analyses across studies, to obtain study results that had not been provided by the trial publications and to check modelling assumptions 3. An important advantage is being able to model individual‐level interactions directly within studies, which has substantially greater power and avoids ecological bias compared with a meta‐regression of aggregate data across studies 4, 5. For such reasons, there has been an increase in the number of IPD meta‐analyses in the last decade 5, 6.

There are two competing statistical approaches for IPD meta‐analysis: a two‐stage or a one‐stage approach 7. In the two‐stage approach, firstly, the IPD from each study are analysed separately in order to obtain aggregate (summary) data of interest (such as an effect estimate and its confidence interval (CI)); then secondly, these are combined by an appropriate fixed‐effect or random effects meta‐analysis model. The alternative one‐stage IPD meta‐analysis approach analyses all the patient‐level data from all the trials in a single step, for example, using a hierarchical (random effects) model that accounts for the clustering of patients within studies 8. The two‐stage approach is often preferred 2, 9 because in the second stage it uses standard meta‐analysis methods that are well documented, for example, in the Cochrane Handbook 10. However, one‐stage methods have also been recommended because they use a more exact likelihood specification 3, 11, which avoids the assumptions of within‐study normality and known within‐study variances, which are especially problematic in meta‐analyses with small studies and/or rare events. Yet, one‐stage methods are also criticised for being computationally intensive and prone to convergence problems 3, 12.

This discrepant advice is causing a dilemma for researchers who are writing grant applications and statistical analysis protocols: do they adopt a one‐stage or a two‐stage approach? This is especially important as statistical and/or clinical conclusions may depend on the chosen approach. For example, Debray *et al.* found that erythema was a statistically significant predictor of deep vein thrombosis (DVT) in a one‐stage analysis (*p* = 0.03), but not a two‐stage analysis (*p* = 0.12) 3. For this reason, Tierney *et al.* advise, ‘It is important, therefore, that the choice of one or two‐stage analysis is specified in advance or that results for both approaches are reported’ 13.

To aid this process, this article provides a tutorial of the two‐stage and one‐stage approaches to IPD meta‐analysis. In Section 2, we introduce the approaches using statistical notation and provide examples for continuous, binary and time‐to‐event outcomes, which illustrate how the two approaches often give similar results. Available statistical software is also summarised. Section 3 then outlines 10 key reasons why differences may arise to help users resolve them if they occur in practice. In particular, we highlight that differences are most likely due to the analyst making discrepant modelling assumptions, changing the specification of unknown parameters or using different techniques for model estimation or CI derivation. Real examples are used to illustrate the messages and include application of fixed‐effect and random effects models for obtaining summary meta‐analysis results for treatment and prognostic effects, treatment‐covariate interactions and test accuracy. Section 4 then concludes with some discussion and recommendations for future IPD meta‐analyses.

## 2 An introduction to one‐stage and two‐stage IPD meta‐analysis models

We now introduce the two approaches using statistical notation, starting with the more familiar two‐stage approach.

### 2.1 The two‐stage approach

#### 2.1.1 First stage

Let us assume that there are *i* = 1 to *K* trials for the IPD meta‐analysis and that a treatment effect is of interest. In the two‐stage approach, the first stage involves a separate analysis in each study to derive the *K* treatment effect estimates and their variances, using an appropriate method chosen by the meta‐analyst. For example, estimates for odds ratios (ORs) or risk ratios (and variances for log ORs and log relative risks) can be derived using standard formulae 14 after collapsing the IPD to 2 × 2 contingency tables. More generally, the estimates and standard errors can be derived by fitting a regression model suitable for the outcome of interest, with a model specification deemed appropriate by the analyst. This enables covariate adjustment if necessary, which is especially important in situations where confounding is a concern. We focus now on utilising familiar regression models for continuous, binary and time‐to‐event outcomes; however, there are many other modelling options available.

If the outcome is continuous (blood pressure, say) then one may use, for example, maximum likelihood (ML) or restricted ML (REML) estimation to fit an appropriate linear regression in each study separately, such as an analysis of covariance (ANCOVA) model. At baseline (i.e. before randomisation) the *j* ^th^ participant in the *i* ^th^ trial provides their initial (blood pressure) value, which we denote by *y*_*Bij*_ (where *B* indicates baseline). Also, each participant provides their final (blood pressure) value after treatment, which we denote by *y*_*Fij*_ (where *F* indicates final). Also, let *x_ij_* be 0/1 for participants in the control/treatment group, respectively. One can then fit the following ANCOVA model to the IPD in each trial separately, where the final score is regressed against the baseline score and the treatment effect:

(1) $$y_{Fij}=\alpha_{i}+\beta_{i}y_{Bij}+\theta_{i}x_{ij}+e_{ij}$$

$$e_{ij}\sim N\left(0,\sigma_{i}^{2}\right)$$

In this model, *α_i_* is the intercept (the expected response in the placebo group for those with a zero *y*_*Bij*_), *θ_i_* is the underlying treatment effect (the mean difference in final score between treatment groups, after adjusting for baseline score), *β_i_* denotes the mean change in *y*_*Fij*_ for a one‐unit increase in *y*_*Bij*_, and *σ_i_* ^2^ is the residual variance of the responses after accounting for the treatment effect and the baseline values.

For non‐continuous outcomes, generalised linear regression models are usually preferred, such as binomial logistic regression for binary outcomes, ordinal logistic regression for ordinal outcomes, multinomial regression for multinomial outcomes and Poisson regression for count outcomes. For example, for a binary outcome (e.g. death by 1 month), one might use ML estimation to fit the following logistic regression model in each trial separately:

(2) $$\ln\left(\frac{E\left(y_{ij}\right)}{1-E\left(y_{ij}\right)}\right)=\ln\left(\frac{p_{ij}}{1-p_{ij}}\right)=\alpha_{i}+\theta_{i}x_{ij}$$

where *y_ij_* is 1 or 0 for participants with or without the outcome, respectively; *p_ij_* is the probability of participant *j* experiencing the event; *α_i_* is the intercept (the expected log odds of the event for the control group); and *θ_i_* denotes the treatment effect (the log OR). Baseline covariates might also be included in equation (2), alongside *x_ij_*, in order to increase power or to adjust for baseline confounding. If interest was in relative risks rather than ORs, then one could fit a binomial regression with a log‐link or a Poisson regression with robust standard errors 15.

If the outcome is time‐to‐event (e.g. time to death), a straightforward approach would be to use ML estimation to fit a Cox regression model in each study separately,

(3) $$h_{ij}\left(t\right)=h_{0i}\left(t\right)exp\left(\theta_{i}x_{ij}\right)$$

where *h*_*ij*_(*t*) is the hazard rate over time, *t*, for participant *j*; *h*_0*i*_(*t*) is the baseline hazard (for those in the placebo group); and *θ_i_* denotes the log hazard ratio (i.e. the treatment effect). As before, baseline covariates might also be included in equation (3), alongside *x_ij_*, in order to increase power or to adjust for baseline confounding.

Following estimation of an equation such as (1), (2) or (3) in each trial separately, the meta‐analyst obtains *K* treatment effect estimates, ${\hat{\theta }}_{i}$, and their variances, Var( ${\hat{\theta }}_{i}$), ready for the second stage (see succeeding paragraphs ). If a different measure is of interest, then the equations should be modified accordingly to allow the measure to be estimated. For example, to examine the interaction between baseline value and treatment effect (a so‐called ‘treatment–covariate interaction’), equation (1) can be modified to

(4) $$y_{Fij}=\alpha_{i}+\beta_{i}y_{Bij}+\theta_{i}x_{ij}+\lambda_{i}\left(y_{Bij}x_{ij}\right)+e_{ij}$$

$$e_{ij}\sim N\left(0,\sigma_{i}^{2}\right)$$

where the interaction term, *λ_i_*, denotes the mean increase in treatment effect for a one‐unit increase in the baseline value. Estimation of equation (4) in each trial then provides the meta‐analyst with *K* treatment–covariate interaction estimates (and their variances) ready for the second stage. In the following subsection, in our description of the second stage, we focus on the synthesis of treatment effects, but the concepts apply equally to meta‐analysis of any parameter estimate of interest.

#### 2.1.2 Second stage

In the second stage, the treatment effect estimates, ${\hat{\theta }}_{i}$, obtained in the first stage are then combined across trials, assuming that the true treatment effects are either fixed or random across studies. The fixed‐effect model assumes that ${\hat{\theta }}_{i}$ are all estimates of the same underlying treatment effect in all studies, represented as *θ*. It can be written generally as 16

(5) $${\hat{\theta }}_{i}\sim N\left(\theta ,Var({\hat{\theta }}_{i})\right)$$

where the Var( ${\hat{\theta }}_{i}$) estimates are also taken from the first stage, and usually assumed known. The most common method to estimate *θ* is the inverse variance method, which provides a weighted average, where the weight of each trial, *w_i_*, is defined as 10

(6) $$w_{i}=\frac{1}{var\left({\hat{\theta }}_{i}\right)}$$

and the pooled treatment effect, *θ*, and its variance are calculated by

(7) $$\hat{\theta }=\frac{\sum_{i=1}^{K}{\hat{\theta }}_{i}w_{i}}{\sum_{i=1}^{K}w_{i}}$$

(8) $$var\left(\hat{\theta }\right)=\frac{1}{\sum_{i=1}^{K}w_{i}}$$

These solutions can also be derived using ML solution estimation. As the fixed‐effect model assumes that the true treatment effect is the same in all studies, the obtained summary estimate, $\hat{\theta }$, should be interpreted as the best estimate of this common treatment effect.

The random effects model allows for between‐study variation, *τ* ^2^, in the true treatment effect and makes the assumption that the different studies are estimating different, yet related, treatment effects. The random effects model can be written generally as 16

(9) $${\hat{\theta }}_{i}\sim N\left(\theta_{i},Var\left({\hat{\theta }}_{i}\right)\right)$$

$$\theta_{i}\sim N\left(\theta ,\tau^{2}\right)$$

where the Var( ${\hat{\theta }}_{i}$) estimates are again assumed known and *u_i_* denotes a random‐effect, which indicates that the treatment effect in the *i* ^th^ trial, *θ_i_*, is assumed normally distributed about an average treatment effect, *θ*, with between‐study variance, *τ* ^2^. As the random effects model assumes the true treatment effect varies across studies, the obtained summary estimate, $\hat{\theta }$, should be interpreted as the estimated *average* of the distribution of true treatment effects in the meta‐analysis. Equation (9) reduces to equation (5) when *τ* ^2^ equals zero.

To obtain meta‐analysis results, an inverse variance approach can again be taken but with the weights of each trial now adjusted to incorporate an estimate of *τ* ^2^:

(10) $$w_{i}^{*}=\frac{1}{var\left({\hat{\theta }}_{i}\right)+{\hat{\tau }}^{2}}$$

Then, the estimate of the pooled effect and its variance are calculated using:

(11) $$\hat{\theta }=\frac{\sum_{i=1}^{K}{\hat{\theta }}_{i}w_{i}^{*}}{\sum_{i=1}^{K}w_{i}^{*}}$$

(12) $$var\left(\hat{\theta }\right)=\frac{1}{\sum_{i=1}^{K}w_{i}^{*}}$$

Perhaps the most popular method of estimating *τ* ^2^ is the non‐iterative, non‐parametric methods of moments (MoM) estimator of DerSimonian and Laird 17, due to its speed and availability in non‐sophisticated statistical packages, such as RevMan 18. It also avoids the assumption of normally distributed effects as written in equation (9), as it simply uses the Q‐statistic and inverse‐variance weights. Other non‐iterative estimators are also available, which have been shown to improve upon DerSimonian and Laird in some situations 19, 20. Other iterative methods are available to estimate *τ* ^2^ and *θ* in equation (9) 19, 21, including ML and REML. Bayesian approaches can also be used, but these are beyond the scope of this paper. Indeed, there is much ongoing debate in the meta‐analysis literature about the best method to estimate *τ* ^2^ and (subsequently) *θ* 22, 23, and we return to this issue in Section 3.

### 2.2 The one‐stage approach

The one‐stage approach analyses all the IPD from all studies simultaneously. The model framework depends on the outcome type and the model specification deemed appropriate by the analyst. In the succeeding discussion, we again focus on specifying typical models for continuous, binary and time‐to‐event outcomes in regard to a summary treatment effect estimate. For survival data, this means we focus mainly on Cox regression models but recognise that other (flexible) parametric survival model specifications are possible. Furthermore, as discussed in the first stage of the two‐stage approach, one‐stage regression models can also be specified for alternative outcomes, such as count and ordinal data.

For continuous outcomes, a one‐stage IPD meta‐analysis can be specified in a linear (mixed) model. For example, assuming a fixed treatment effect, the following one‐stage ANCOVA model can be fitted to all studies simultaneously:

(13) $$y_{Fij}=\alpha_{i}+\beta_{i}y_{Bij}+\theta x_{ij}+e_{ij}$$

$$e_{ij}\sim N\left(0,\sigma_{i}^{2}\right)$$

The parameters are as defined previously, but we emphasise that the subscript, *i*, denotes that a separate parameter is estimated for each study. For example, *α*_*i*_ denotes that a separate intercept term is estimated for each study; this is sometimes referred to as ‘stratifying by trial’, and it allows for different mean control group responses per trial and thus accounts for the clustering of participants in trials. Similarly, *β*_*i*_ denotes that each trial has a different adjustment term for baseline values, and $\sigma_{i}^{2}$ denotes a distinct residual variance per trial. It is also important to note that the assumptions of distinct baseline values and residual variances represent just one possible model. Other options are available, and they will be discussed in this article as possible reasons for differences between the one‐stage and two‐stage approaches.

Equation (13) could be extended to include a random treatment effect:

(14) $$y_{Fij}=\alpha_{i}+\beta_{i}y_{Bij}+\theta_{i}x_{ij}+e_{ij}$$

$$\theta_{i}=\theta +u_{i}$$

$$u_{i}\sim N\left(0,\tau^{2}\right)$$

$$e_{ij}\sim N\left(0,\sigma_{i}^{2}\right)$$

Some researchers also prefer to place a random effect on the study intercepts 24; for example equation (13) is then modified to

(15) $$y_{\text{Fij}}=\alpha_{i}+\beta_{i}y_{\text{Bij}}+\theta x_{\text{ij}}+e_{\text{ij}}$$

$$\alpha_{i}\sim N\left(\alpha ,\delta^{2}\right)$$

$$e_{ij}\sim N\left(0,\sigma_{i}^{2}\right)$$

This is potentially a strong assumption, and will usually be unnecessary, but illustrates the so‐called flexibility of the one‐stage approach 3. It is perhaps more realistic and most helpful when the average baseline response *α* is also of interest, or when needing to reduce (perhaps due to convergence problems) the number of parameters to estimate (that is, rather than estimating *K* intercepts in equation (13), we now estimate just *α* and *δ* in equation (15)). A similar modification can be made to equation (14) to include a random treatment effect and a random study effect, with a suitable correlation structure between the random effects. Other ‘flexible’ modifications are also possible; for example, assuming each study has a common (rather than separate) baseline adjustment term and a common (rather than separate) residual variance 25. As mentioned, we return to these issues in Section 3. The aforementioned linear (mixed) models are typically estimated using ML or REML.

For binary outcomes, a generalised linear (mixed) model is required. For example, a one‐stage logistic regression meta‐analysis, with stratified intercepts and a random treatment effect, can be expressed as

(16) $$\ln\left(\frac{E\left(y_{ij}\right)}{1-E\left(y_{ij}\right)}\right)=\ln\left(\frac{p_{ij}}{1-p_{ij}}\right)=\alpha_{i}+\theta_{i}x_{ij}$$

$$\theta_{i}=\theta +u_{i}$$

$$u_{i}\sim N\left(0,\tau^{2}\right)$$

where model parameters are as defined previously. As for the one‐stage general linear model, the distinct intercept term per trial accounts for different baseline (control group) risks and the clustering of participants in each trial. Alternative specifications of equation (16) could be chosen, including a fixed rather than random treatment effect, and a random rather than stratified trial‐specific intercept term. Adjustment terms could also be included, and then the analyst must decide whether a common or distinct adjustment term is specified per trial. Estimation is typically performed using ML via a numerical approach such as Gaussian quadrature.

For time‐to‐event outcomes, a one‐stage Cox regression model can be used 9, 12, 26, 27. However, unlike the continuous and binary outcome frameworks, there is the added complexity of whether to specify unique or proportional baseline hazards for the trials. For example, assuming a random treatment effect, one might specify proportional baseline hazards by adjusting for a study‐specific effect:

(17) $$h_{ij}\left(t\right)=h_{0}\left(t\right)\exp\left(\alpha_{0i}+\theta_{i}x_{ij}\right)$$

$$\theta_{i}=\theta +u_{i}$$

$$u_{i}\sim N\left(0,\tau^{2}\right)$$

Here, *h*_0_(*t*) is the baseline hazard function in the reference trial (say *i* = 1), and *α*_0*i*_ is the proportional effect on the baseline hazard function due to the *i^th^* trial (with *α*_01_ constrained to be zero). Alternatively, we can specify unique baseline hazard functions for each trial (i.e. stratify by trial) by

(18) $$h_{ij}\left(t\right)=h_{0i}\left(t\right)\exp\left(\theta_{i}x_{ij}\right)$$

$$\theta_{i}=\theta +u_{i}$$

$$u_{i}\sim N\left(0,\tau^{2}\right)$$

where *h*_0*i*_(*t*) is the unique baseline hazard function in the *i^th^* trial. By avoiding the assumption of proportional baseline hazards, equation (18) makes less assumptions than equation (17). As for linear and logistic models, the flexibility of the one‐stage approach also allows the analyst to make other modifications if desired, including a fixed rather than random treatment effect, a random rather than stratified trial‐specific intercept term (for equation (18)) and the inclusion of common adjustment terms.

Estimation of such one‐stage Cox models can be via ML or REML 9, which typically avoid estimation of the baseline hazard itself 1, 28, 29, 30. Crowther *et al.* also show how to fit the models in a Poisson regression framework using ML estimation 26. If the baseline hazards are themselves of interest (for example, for developing a prognostic model 31, 32), a one‐stage parametric survival model might be preferred; for example, Crowther *et al.* use Gauss–Hermite quadrature to estimate a one‐stage IPD meta‐analysis model akin to equation (18), but using a flexible parametric approach where the baseline cumulative hazard is modelled via restricted cubic splines 33.

### 2.3 Statistical software

Debray *et al.* provide an excellent overview of statistical software for IPD meta‐analysis 1. They focus mainly on one‐stage models and highlight software that fits the necessary generalised linear mixed models, such as SAS 34, Stata 35, R 36 and MLwiN 37. We additionally wish to highlight the ‘ipdforest’ module in Stata, which produces a forest plot following a one‐stage IPD meta‐analysis of a continuous or binary outcome. This gives the one‐stage summary result at the bottom of the plot, but with the study‐specific estimates as derived from the first stage of a two‐stage analysis 38. Debray *et al.* also do not mention the excellent ‘ipdmetan’ package 39; a Stata module that automatically operates the first and second stages of the two‐stage approach. This can handle any outcome type (including continuous, binary or time‐to‐event outcomes) in the first stage and offer a wide variety of estimation options for the second stage, whilst also producing detailed summary results, heterogeneity statistics and a forest plot. Several modules and software, such as ‘metan’ 40 in Stata, ‘meta’ and ‘metafor’ in R 36, 41 and RevMan 18 can perform the first stage of a two‐stage meta‐analysis if the user provides the 2 × 2 contingency tables. If users are happy to implement the first stage themselves, then there are also a plethora of other modules and software that can implement the second stage directly, including ‘metan’ 40, ‘metaan’ 42, ‘metareg’ 43 and ‘mvmeta’ 44 in Stata 45, ‘meta’ and ‘metafor’ in R 36, 41, SAS Proc Mixed 46, 47 and RevMan 18. However, the available estimation methods differ considerably across these.

### 2.4 One‐stage and two‐stage approaches often give very similar results

Several authors have investigated the difference between one‐stage and two‐stage IPD meta‐analysis results (for example, see the following 1, 2, 3, 9, 48, 49, 50), either empirically, theoretically or via simulation. Most authors conclude that they give very similar results. For example, for binary outcomes, Stewart *et al.* state,

Major benefits of obtaining IPD are accrued prior to analysis and where an IPD review evaluates effectiveness based on sufficient data from randomised controlled trials, one‐stage statistical analyses may not add much value to simpler two‐stage approaches. Researchers should therefore not be discouraged from undertaking IPD synthesis through lack of advanced statistical support 2.

Debray *et al.* also investigated any differences in relation to binary outcomes 3 and concluded that generally, the approaches gives similar results, but all have potential estimation challenges. Also, Senn (2010) discussed the theoretical equivalence of one‐stage and two‐stage likelihood specifications for binary fixed effects meta‐analysis without covariates 51. Similarly, for time‐to‐event outcomes, Bowden *et al.* conclude that if the aim of a meta‐analysis is to estimate the treatment effect under a random effects model, there appears to be only a very small gain in fitting more complex and computationally intensive one‐stage models 9. For continuous outcomes, Mathew and Nordstrom (2010) extend previous theoretical work to show that one‐stage and two‐stage summary estimates coincide exactly when fixed treatment and fixed intercept terms are used 52, 53, 54.

Two recent overviews of IPD meta‐analysis also note that one‐stage and two‐stage approaches usually give very similar results 3, 13. To illustrate this, Tierney *et al.* give a binary outcome example where the effects of anti‐platelets on pre‐eclampsia in pregnancy from two‐stage (relative risk = 0.90, 95% CI = 0.83 to 0.96) and one‐stage (relative risk = 0.90, 95% CI = 0.83 to 0.97) analyses were almost identical 2, 13.

**Figure 1:**
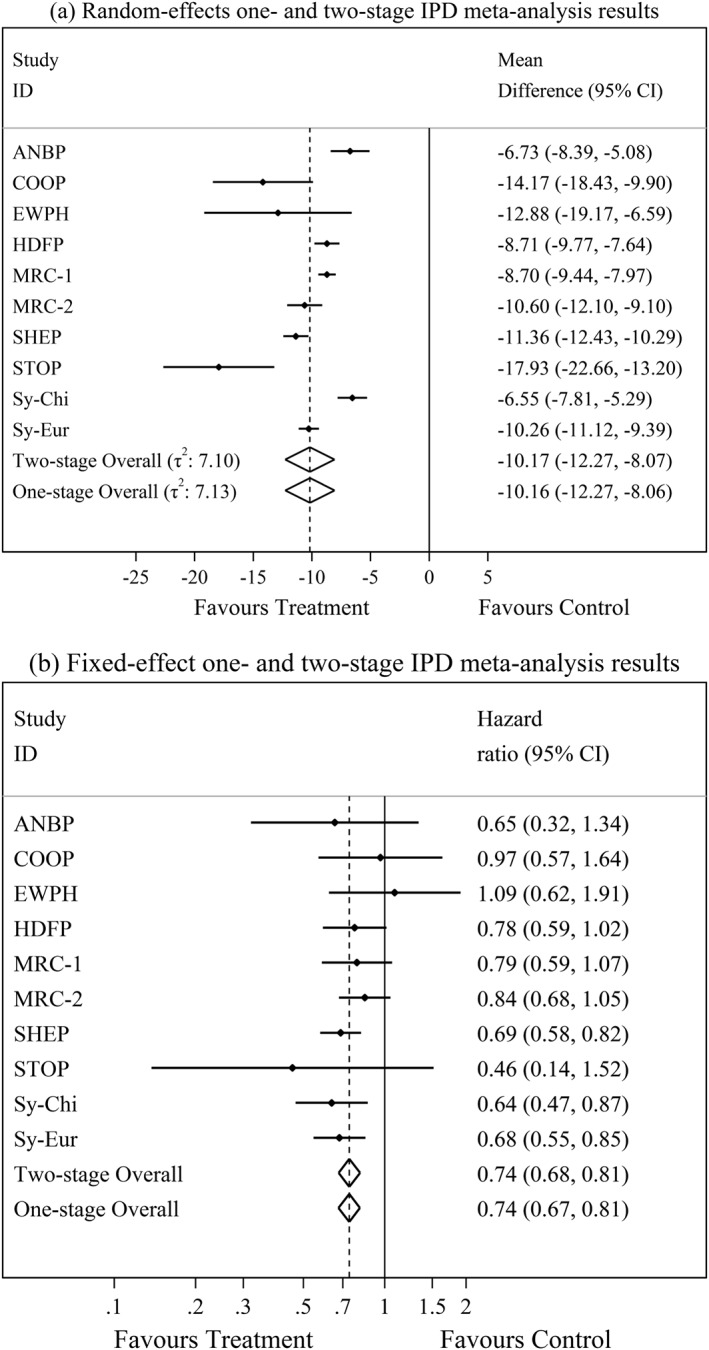
One‐stage and two‐stage IPD meta‐analysis summary treatment effect results for (a) mean difference for final systolic blood pressure adjusting for baseline systolic blood pressure and (b) hazard ratio for cardiovascular disease. IPD, individual participant data; CI, confidence interval.

Figure 1(a) gives a continuous outcome example, from a random effects IPD meta‐analysis of 10 randomised trials to evaluate the effect of anti‐hypertensive treatment on systolic blood pressure 55. Again, the summary results are almost identical when using either the one‐stage (equation (14)) or two‐stage (equation (1) followed by equation (9)) approaches using REML estimation. Figure 1(b) gives a time‐to‐event example, showing a fixed‐effect IPD meta‐analysis of the same 10 hypertension trials 26. Again, the summary treatment effect on cardiovascular disease is very similar when using either a two‐stage approach (equation (3) followed by (5)), or a stratified one‐stage approach with a unique baseline hazard per study (equation (18)), via ML estimation.

## 3 Key reasons why meta‐analysis results may differ for the one‐stage and two‐stage approaches

The examples in Section 2.4 echo the wide‐spread belief that one‐stage and two‐stage results are usually very similar. However, differences can arise 53, and sometimes, these may even be large with discrepant statistical or clinical significance 3.

To aid researchers facing this situation, we now describe 10 key reasons why such differences may arise even when the same IPD are used for both one‐stage and two‐stage approaches. These relate to different modelling assumptions, parameter specifications and estimation methods. Although some are perhaps obvious, the majority are subtle, and we suspect that most researchers are unaware of their potential impact. We illustrate each reason with results from previous IPD meta‐analyses. In all examples, we ensure that exactly the same IPD are used for both one‐stage and two‐stage analyses, and thus, any differences cannot be due to discrepant numbers of studies, patients or follow‐up times. Some of the issues are inter‐related, but we highlight them separately to make them explicit.

### 3.1 Reason I: exact one‐stage likelihood versus approximate two‐stage likelihoods

One‐stage and two‐stage methods may yield different summary results when the second stage of the two‐stage method assumes that study treatment effect estimates ( ${\hat{\theta }}_{i}$) have a normal sampling distribution and that their variances ( $Var\left({\hat{\theta }}_{i}\right))$ are known 3, 11, 56, 57. This first assumption is based on the central limit theorem, and the second assumes that the variance is estimated with reasonable accuracy. These assumptions depend on a combination of the total number of participants in each trial, the number of participants in each treatment group in each trial and the number of events/non‐events in each group in each trial. Therefore, both assumptions are unlikely to be appropriate for all outcome types when some or many of the included studies are small (<30 participants). However, if the small trials do not contribute greatly to the pooled effect (i.e. the weight of these trials is low), then this is a less concerning issue. The assumptions are more generally unreliable for binary and time‐to‐event outcomes when outcomes are rare (or extremely common), as the number of outcomes (and non‐outcomes) then drives the study‐specific estimates and their variances, not just the total participants. In contrast, a one‐stage IPD meta‐analysis model, such as a logistic regression model in equation (16), directly models the actual distribution of the IPD (e.g. binomial for logistic regression) and avoids making any assumptions about the distribution of the treatment effect estimates in each study. It thereby provides a more exact likelihood specification from which to make inferences 11, 58. The one‐stage approach may additionally, but perhaps more critically, be different from the two‐stage approach if there are zero event counts for binary outcomes because the two stage method typically requires a continuity correction (such as +0.5 to cells in the available two‐by‐two tables 59, 60, 61), whereas this is not necessary with the one‐stage logistic regression model 11. The issue that effect estimates are not normally distributed is possibly less of a concern than the issue of zero cells and subsequent continuity corrections, as the latter actually influence the magnitude of effect estimates and their estimated variances, which may introduce bias that then feeds in to the second stage.

**Figure 2:**
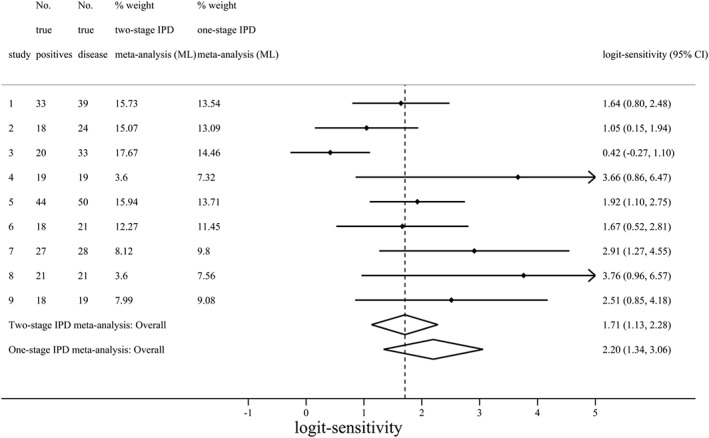
Forest plot of the one‐stage and two‐stage meta‐analysis results for the sensitivity of the PET test for diagnosis of Alzheimer's disease. PET, positron emission tomography; IPD, individual participant data; ML, maximum likelihood.

We illustrate this issue with an example from Hamza *et al.* 11 who re‐analysed an IPD meta‐analysis of nine studies that assessed the operating characteristics of positron emission tomography in the diagnosis of Alzheimer's disease. There were small numbers of patients in each study (range: 19–50) with a total of 254 diseased patients and 218 true positive test results, and the aim was to summarise the sensitivity of the positron emission tomography test. Although sensitivity and specificity are often analysed together, here, we just consider sensitivity alone; indeed, a joint analysis often makes little difference anyway 62, 63. However, three studies contained zero false positives (sensitivity = 100%), and therefore, to perform the two‐stage approach, Hamza *et al.* used continuity corrections (+0.5 added to each cell) in the first stage to derive logit‐sensitivity estimates and their variances in each study (equation (2)). In contrast, the one‐stage approach does not require such assumptions as it models the binomial distribution of the IPD directly (here, a logistic regression model with just an intercept term denoting the logit‐sensitivity and an associated random effect). This leads to large differences in the summary logit‐sensitivity estimate between the one‐stage (ML estimate: 2.20) and two‐stage (ML estimate: 1.71) approaches, relating to a summary sensitivity of 0.90 and 0.85, respectively. The estimated *τ* ^2^ was also different (0.97 in one‐stage analysis, 0.37 in two‐stage analysis), which may be especially important for making predictive inferences about the sensitivity in a new population 64. Riley *et al.* propose how to derive percentage study weights for this example, and these are shown in Figure 2, with clear differences in the one‐stage and two‐stage weights (Riley *et al.*, *submitted*).

### 3.2 Reason II: likelihood‐based one‐stage versus alternative weighting schemes in two‐stage

As described in Reason I, stage 2 in a two‐stage approach is problematic when outcomes are rare and studies are small and may even be problematic when some studies have unbalanced sample size in the treatment and control group numbers 10, 59, 60. To address this, alternative two‐stage approaches have been proposed, which use a different weighting scheme to the inverse variance method, such as the Peto method 65 and the Mantel–Haenszel method 66, which also avoid the use of continuity corrections when there are zero cells. The Peto method is considered to work well when intervention effects are small, event risks are <1%, and when there are balanced experimental and control group sizes within trials 10, 60. The Mantel–Haenszel is often preferred when event risk is >1%, and there are unbalanced data 10.

However, in the context of this article, it is important to emphasise that even these alternative two‐stage methods may give different results to a one‐stage meta‐analysis that uses the binomial likelihood, such as a logistic regression. We illustrate this issue with a previous IPD meta‐analysis dataset of three trials that investigated whether erythema is a risk factor for deep vein thrombosis (DVT), as shown in Debray *et al.* 3. In terms of a two‐stage approach, the Mantel–Haenszel method might be preferred in this situation because the event rates are not small (10–29%), and sample sizes are not balanced in the control and treatment groups in all three trials. Assuming a fixed treatment effect, the summary results from the Mantel–Haenszel and Peto methods are compared with a one‐stage logistic regression model (equation (16) with a fixed treatment effect) in Table I.

**Table I:** Summary results from a fixed‐effect IPD meta‐analysis of the erythema data using two‐stage or one‐stage approaches.

Approach	Method	Summary OR (95% CI), *p*‐value
Two‐stage	Mantel–Haenszel	1.277 (0.980 to 1.665), 0.070
Two‐stage	Peto	1.285 (0.980 to 1.686), 0.069
One‐stage	Logistic regression	1.352 (1.031 to 1.771), 0.029

IPD, individual participant data; OR, odds ratio; CI, confidence interval.

Although summary results are qualitatively similar, the summary OR estimates are slightly lower for the two‐stage methods compared with the one‐stage approach. Furthermore, only the one‐stage approach suggests a statistically significant increase in the odds of DVT if there is erythema compared with without. These differences are a consequence of the different weighting schemes employed by the methods.

### 3.3 Reason III: clustering and choice of specification for the intercept

A two‐stage IPD meta‐analysis automatically accounts for clustering of patients by trial by analysing the data from each trial separately in the first stage. However, a one‐stage IPD meta‐analysis models all data simultaneously, and therefore, the analyst must account for the clustering of patients to avoid misleading effect estimates and conclusions. More specifically, for logistic regression models, one expects downwardly biassed results when ignoring the clustering due to non‐collapsibility of the OR 8. As discussed in Section 2.2, the clustering can be accounted for by stratifying the analysis by trial (i.e. estimating a separate intercept or baseline hazard for each trial) or assuming that the intercept (baseline hazard) is randomly drawn from some distribution. However, there is repeated evidence that some researchers are ignoring the clustering of patients within trials in the one‐stage IPD meta‐analyses and are therefore analysing data as if it were coming from a single study 7, 8, 67. This is another key reason why one‐stage and two‐stage results may differ.

We illustrate this issue using a previous IPD meta‐analysis dataset of two randomised controlled trials (total patients = 1620) that investigated whether nicotine gum increased the odds of smoking cessation 68. All parameter estimates are obtained using ML for consistency and assume a fixed treatment effect (Table II). The two‐stage approach used equation (2) followed by equation (5), and thus, the first‐stage automatically assumed a different intercept term per trial. The one‐stage approach that accounted for clustering used equation (16) with a separate intercept per trial and a fixed treatment effect; however, the one‐stage approach that ignored clustering used the same equation, but with just a single intercept term.

**Table II:** Fixed‐effect one‐stage and two‐stage approaches to illustrate the effect of clustering in the nicotine gum dataset 68.

Approach	Clustering	Summary OR (95% CI OR)
Two‐stage	Accounting for clustering	1.769 (1.257 to 2.488)
One‐stage	Ignoring clustering	1.398 (1.020 to 1.916)
One‐stage	Accounting for clustering	1.802 (1.290 to 2.517)

OR, odds ratio; CI, confidence interval; *τ* ^2^, between‐study heterogeneity.

The results highlight the importance of accounting for clustering. The summary OR estimate and CI from the one‐stage approach ignoring clustering only just suggest that there is a statistically significant association between nicotine gum use and smoking cessation. However, in contrast, the summary OR estimate from both the two‐stage and one‐stage approaches that account for clustering are substantially higher, and there is much stronger statistical evidence that nicotine gum is beneficial.

Thus, clustering must be accounted for; yet, even the choice of *how* the clustering is accounted for may cause differences. For example, for continuous outcomes, Matthew and Nordstrom (2010) suggest that a one‐stage approach with a random intercept term may be slightly more precise than a two‐stage IPD meta‐analysis (which has a distinct intercept term per study) 53. For time‐to‐event outcomes, there are even more options. In a two‐stage IPD meta‐analysis, the baseline hazard is uniquely estimated in each trial separately, and thus, there is no assumption about how the shape or magnitude of the baseline hazard is related across trials. In a one‐stage meta‐analysis, the analyst could also assume this (equation (18)) but, due to the greater flexibility of the approach, could alternatively assume that the baseline hazards are distinct but proportional to each other (as in equation (17)) or assume a random effect (frailty) for the baseline hazard; for example, the ‘shared’ option within the ‘stcox’ module within Stata treats the frailties as being gamma distributed (mainly for computational convenience) 35. In our experience, this decision usually only leads to small differences in the summary treatment effect. For example, in an IPD meta‐analysis of 1225 patients from five clinical trials in epilepsy that investigated the effect of treatment on time to remission 29, a one‐stage IPD meta‐analysis Cox model with proportional baseline hazards (equation (17)) gave a summary hazard ratio of 0.89 (95% CI: 0.77 to 1.03, *p* = 0.115). The summary hazard ratio was 0.90 (95% CI: 0.78 to 1.03, *p* = 0.130) in a Cox model with unique baseline hazard functions for each trial, and 0.90 (95% CI: 0.78 to 1.04, *p* = 0.153) assuming a frailty model. The differences are qualitatively small; nevertheless, changes in the *p*‐value are still apparent and may be more pertinent in other examples.

### 3.4 Reason IV: choice of specification for any adjustment terms

Similar to the choice of intercept (baseline hazard) specification, in a one‐stage IPD meta‐analysis, the analyst can also adopt different specifications for any adjustment terms (such as adjustment for baseline score in an ANCOVA, equation (13)). A two‐stage approach automatically assumes a different effect of each adjustment factor in each trial, as it analyses each trial separately in the first stage (equation (1)). A one‐stage approach can replicate this by stratifying the effect by study (as in equation (13)), but alternatively, the analyst could assume that the effects were random or even fixed. The fixed assumption is likely an over‐simplification, but our experience suggests that it is often adopted in practice, perhaps unknowingly.

The choice of specification of adjustment factors may lead to differences between the one‐stage and two‐stage approaches, particularly if the effect of the adjustment factor is heterogeneous across the trials. When there are multiple random effects, the specification of their covariance matrix (e.g. unstructured or independent) may also be influential.

We illustrate this using an IPD meta‐analysis by Riley *et al.* 55, which used the 10 hypertension trials introduced in Section 2.4. Here, instead of focussing on the treatment effect, the objective was to examine whether smoking is a prognostic factor for high systolic blood pressure at follow‐up, after adjusting for baseline blood pressure and treatment group. Utilising a one‐stage linear regression model (similar to equation (14) with an additional term for smoking), we compare REML results for the smoking effect when including baseline blood pressure and treatment group as (a) fixed, (b) random with an unstructured covariance structure, (c) random with an independent covariance structure or (d) distinct adjustment terms for each trial. All models assumed a random effect for smoking. The competing two‐stage approach used a linear regression model (similar to equation (1) with additional term for smoking and parameters estimated) in each trial separately with smoking status, baseline blood pressure and treatment group, and then a random effects meta‐analysis model (equation (9)) using REML to synthesise the smoking effect in the second stage.

Table III shows that the estimate and 95% CI of the summary effect of smoking differs depending on how the adjustment terms were accounted for, although all approaches suggest smoking is a prognostic factor. The one‐stage approach with distinct adjustment terms is very similar to the two‐stage approach, as expected as the two‐stage approach also makes the same assumption. However, the one‐stage approach with fixed or random adjustment terms (especially with an unstructured covariance matrix) gives a lower prognostic effect and a wider CI.

**Table III:** One‐stage and two‐stage REML results for the effect of smoking on blood pressure at follow‐up after different specifications of the adjustment for baseline blood pressure and treatment group.

Approach	Model specification in regard to adjustment factors	Summary mean difference (smokers versus non‐smokers), 95% CI	$\hat{\tau }$ ^2^
Two‐stage	Distinct per trial	1.763 (1.146 to 2.380)	0.227
One‐stage	Fixed	1.689 (0.951 to 2.426)	0.271
One‐stage	Random (correlated*)	1.523 (0.731 to 2.316)	0.510
One‐stage	Random (independent*)	1.744 (1.027 to 2.461)	0.235
One‐stage	Distinct per trial	1.756 (1.043 to 2.469)	0.229

* Unstructured or independent covariance structure for all included random‐effects.

REML, restricted maximum likelihood; CI, confidence interval; *τ* ^2^, between‐study heterogeneity in the smoking effect.

### 3.5 Reason V: choice of specification for the residual variances

For continuous outcomes, in the two‐stage approach, the residual variances are automatically distinct in each trial, as separate linear models are applied to each trial (equation (1)). In a one‐stage IPD meta‐analysis, such as in equation (14), the residual variance can also be allowed to vary by trial, such that *e_ij_* ~ N(0, *σ_i_* ^2^). However, it is possible to simplify this and make an additional assumption that all trials have the same residual variance, such that *e_ij_* ~ N(0, *σ* ^2^). In our experience, many one‐stage analysts are not aware that they even make this assumption, but it can cause summary results to differ to those from the two‐stage approach. This is illustrated using the IPD meta‐analysis of hypertension trials again (introduced in Section 2.4).

When we assume different residual variances for each trial, the one‐stage and two‐stage results are almost identical (Table IV). However, the one‐stage analysis that assumes the same residual variance in each trial gives a slightly larger summary result, a larger estimate of *τ* ^2^ and wider CIs. The larger ${\hat{\tau }}^{2}$ is caused by the mis‐specified residual variances. Although clinical conclusions are qualitatively the same, this illustrates how the specification of the residual variances can potentially cause differences in meta‐analysis estimates. For multivariate responses (e.g. multiple time points), a related issue is whether the analyses allow for separate or common residual variances for each endpoint, or separate or common correlations between endpoints (Reason IX).

**Table IV:** One‐stage and two‐stage REML results for the effect of hypertension treatment on systolic blood pressure, after different specifications of the residual variances.

Approach	Assumption of residual variances in the trials	Summary mean difference (95% CI)	${\hat{\tau }}^{2}$
One‐stage	Same in each trial	−10.34 (−12.55 to −8.13)	8.19
One‐stage	Distinct per trial	−10.16 (−12.27 to −8.06)	7.13
Two‐stage	Distinct per trial	−10.17 (−12.27 to −8.07)	7.10

REML, restricted maximum likelihood; CI, confidence interval; *τ* ^2^, between‐study heterogeneity in the treatment effect.

### 3.6 Reason VI: choice of fixed‐effect or random effects for the parameter of interest

Perhaps the most obvious explanation for any differences in one‐stage or two‐stage approaches is that, for the parameter of interest, the meta‐analyst may be assuming a fixed‐effect in one approach and random effects in the other. For example, using the hypertension dataset again, we fitted an ANCOVA model to evaluate the effect of hypertension treatment on blood pressure, with a fixed treatment effect in a one‐stage approach (equation (13)), and a random treatment effect in a two‐stage approach (equation (1) followed by equation (9)). The summary treatment effect in the one‐stage approach was −9.31 (95% CI: −9.70 to −8.92) compared with −10.17 (95% CI: −11.99 to −8.35) in the two‐stage approach, with the lower effect in the one‐stage approach due to the fixed‐effect assumption. Sometimes, a fixed‐effect assumption may be enforced when using the one‐stage approach, due to convergence problems (especially when the outcome is rare 3) or computational time (especially for time‐to‐event outcomes with large datasets 12), thus preventing a direct comparison with a two‐stage random effects analysis.

### 3.7 Reason VII: different estimation method for *τ* ^2^

There are various estimation methods for the between‐study heterogeneity parameter, *τ* ^2^, as mentioned in Section 2. However, some are only available in a two‐stage approach and others in only the one‐stage approach. Furthermore, some estimation methods may fail to converge because of small numbers of studies in the meta‐analysis or large within‐study variances 3. Therefore, differences in one‐stage and two‐stage meta‐analysis results may be a consequence of different estimation methods. For example, it is well known that the ML method tends to underestimate the between‐study heterogeneity 69, 70, 71, 72, 73, 74 and that REML is preferred in comparison. However, for non‐continuous outcomes, one‐stage models typically use ML estimation as it is usually the only option. Furthermore, the MoM estimator of DerSimonian and Laird is still the most common estimation method applied in the two‐stage approach and is the only option available in RevMan, for example.

We now compare the IPD meta‐analysis results for the 10 hypertension trials once more, according to different estimation methods. For the two‐stage approach, we fitted model (1) and then adopted a random effects model (9), which was estimated using either MoM, ML or REML. In a one‐stage model, ANCOVA model (14) was fitted, and the model parameters are estimated using REML or ML. The results are shown in Table V.

**Table V:** Summary treatment effect results for the hypertension data to illustrate the differences in summary results according to the estimation method.

Approach	Estimation method	Summary mean difference (95% CI)	${\hat{\tau }}^{2}$
Two‐stage	MoM	−9.85 (−11.13 to −8.57)	3.07
Two‐stage	ML	−10.10 (−12.03 to −8.16)	5.84
Two‐stage	REML	−10.17 (−12.27 to −8.07)	7.10
One‐stage	ML	−10.03 (−11.83 to −8.23)	4.94
One‐stage	REML	−10.16 (−12.27 to −8.06)	7.13

CI, confidence interval; MoM, method‐of‐moments; ML, maximum likelihood; REML, restricted maximum likelihood; *τ* ^2^, between‐study heterogeneity in the treatment effect.

Although summary treatment effect estimates are broadly similar regardless of the estimation approach, it is notable that the estimate of the between‐study variance is substantially affected. For example, ${\hat{\tau }}^{2}$ is much smaller with the MoM estimator in a two‐stage approach ( ${\hat{\tau }}^{2}$ = 3.07) compared with the estimate from a one‐stage approach via REML ( ${\hat{\tau }}^{2}$ = 7.13). Larger ${\hat{\tau }}^{2}$ estimates lead to wider 95% CIs for the summary treatment effect and would have even more impact upon predictive inferences, such as 95% prediction intervals 75. If the same estimation method is used for a one‐stage or two‐stage approach, the summary estimates and 95% CI are very similar. The choice of best estimator for ${\hat{\tau }}^{2}$ is an ongoing issue in the meta‐analysis field 21.

### 3.8 Reason VIII: derivation of CIs

Not only are there competing estimation methods for fitting the random effects meta‐analysis model, but there are also competing methods for the derivation of 95% CIs for the summary effect *post*‐estimation. Standard CIs based on the normal distribution (calculated using the summary estimate ± 1.96 × s.e. of the summary estimate) are often too narrow, especially because ${\hat{\tau }}^{2}$ is often underestimated and no account is taken of the additional uncertainty in ${\hat{\tau }}^{2}$ 22, 76. Therefore, alternative methods have been proposed for deriving 95% CIs in the two‐stage approach, such as the Hartung–Knapp–Sidik‐Jonkman (HKSJ) modification to the variance of the summary estimate 77, 78, 79, 80, 81, combined with the *t*‐distribution with *k* − 1 (*k* = number of studies) degrees of freedom (rather than 1.96 from the standard normal distribution). In a one‐stage approach, the standard approach is again to use the normal distribution from which large sample inferences are based. However, there are also various methods for small‐sample inference based on the *t*‐distribution, also known as denominator‐degrees‐of‐freedom adjustments, including Satterthwaite and Kenward‐Roger 82.

Therefore, differences in 95% CIs from one‐stage and two‐stage approaches may simply be due to the different derivation methods embedded in software packages (e.g. the standard approach is the default in the Stata package ‘metan’ 40, but the HKSJ is the default in the Stata package ‘metareg’ 43). To illustrate this, we return again to the hypertension example. Figure 1 already showed how standard CIs for the summary treatment effect were almost identical for one‐stage and two‐stage analyses (−12.27 to −8.07). However, the 95% CI is slightly wider (−12.44 to −7.90) in a two‐stage approach using the HKSJ variance estimator and *t*‐distribution.

### 3.9 Reason IX: accounting for correlation amongst parameters

A one‐stage approach automatically accounts for all correlation amongst included parameters during model estimation. However, a standard two‐stage approach (as outlined in Section 2) does not account for such correlations unless a multivariate meta‐analysis model is used to jointly synthesise all parameters, whilst accounting for their within‐study, and possibly between‐study, correlations 55, 83. This could lead to the standard two‐stage approach having reduced efficiency and even a change in the summary estimates. Sometimes, even if desired, the multivariate model may not be estimable, especially when there are many parameters to jointly synthesise, because of the lack of information to estimate the between‐study variance–covariance matrix (particularly the unstructured option).

Consider a meta‐analysis of longitudinal outcomes, where each patient in a trial provides an outcome value at each of multiple time points during follow‐up. These patients responses, and indeed subsequent parameter estimates (such as treatment effects at multiple time points), will be correlated, and ignoring this in the meta‐analysis is inadvisable 84, 85. Usually, there are missing data in such situations, such that not all patients provide all time points; furthermore, not all studies will measure the same time points. In a one‐stage analysis of all time points, under a missing at random assumption, even patients and studies with missing time points will still contribute towards the estimation of parameters at *all* time points, by utilising the correlation amongst them. However, a standard univariate two‐stage analysis will typically take each time point separately, and thus exclude patients or studies at particular time points if they did not provide data.

An example of this was illustrated by Jones *et al.* using an IPD meta‐analysis of longitudinal data from five trials that investigated Selegiline versus placebo for the treatment of Alzheimer's disease 84, 86. The outcome of interest over time was the mini‐mental state examination, which is a measure of cognitive function. Patients were repeatedly followed up over time at 1, 2, 4, 6, and 12 months; however, the set of time points available in each trial was different, and not all patients provided all time points in the same trial. The one‐stage approach applied a linear regression model including a separate intercept, baseline adjustment term, and residual variance for each trial, and a fixed treatment effect assumed at each distinct time point. This approach accounts for the correlation amongst patient responses with correlated residuals, and handles missing time point data for participants and trials via a missing at random assumption. The two‐stage approach firstly analysed each trial separately accounting for correlated patient responses within each trial, to produce a treatment effect at each time point reported; however, in the second stage, each time point was analysed separately (independently), ignoring correlation amongst time points, and thus studies only contributed towards the time points they reported.

There are large differences in the summary treatment effects and their standard errors for the two‐stage and one‐stage approaches (Table VI). For example, at time point 4 months, the treatment effect is 0.34 (s.e. = 0.48) for the one‐stage model, compared with 0.75 (s.e. = 0.57) for the two‐stage model. Differences are due to the one‐stage approach utilising the correlation amongst time points, which allows the ‘borrowing of strength’ from the available data to inform the missing data and increases precision 87. When a multivariate model is used in the second stage of the two‐stage approach to account for the correlation, the results become almost identical to those from the one‐stage method 84.

**Table VI:** ML results of one‐stage and two‐stage meta‐analysis of MMSE longitudinal data: estimates (standard error) of difference between Selegiline and Placebo.

Time point	One‐stage model	Standard two‐stage approach (each time point assumed independent)	Multivariate two‐stage approach (correlation amongst time points accounted for)
1 month	0.31 (0.47)	0.43 (0.54)	0.30 (0.47)
2 months	−0.48 (0.62)	−0.84 (0.97)	−0.47 (0.59)
4 months	0.34 (0.48)	0.75 (0.57)	0.33 (0.47)
6 months	0.20 (0.49)	0.31 (0.50)	0.19 (0.48)
9 months	0.35 (0.53)	0.69 (0.63)	0.34 (0.52)
12 months	−0.02 (0.56)	0.29 (0.66)	−0.03 (0.55)

ML, maximum likelihood; MMSE, mini‐mental state examination.

### 3.10 Reason X: ecological bias for treatment covariate interactions

When one wishes to model treatment–covariate interactions, it is important to avoid ecological bias, which occurs when across‐study associations (between mean covariate values and treatment effects across trials) do not reflect the true within‐study relationships (between individual covariate values and individual response to treatment) 4, 88. For example, when looking at the interaction between sex and treatment effect, across‐study associations would allow studies with only males (or only females) to be included, despite containing no within‐study differences between males and females. We note that a similar problem would occur if one used data from single arm trials in a meta‐analysis to inform the estimate of overall treatment effect 51.

A two‐stage approach to the estimation of treatment–covariate interactions automatically avoids ecological bias by fitting a separate model (such as equation (4)) in each trial to obtain within‐study interaction estimates, which are then synthesised in the second stage. Thus any across‐study associations are ignored. This can be replicated in a one‐stage approach by separating the within‐study and across‐study interaction effects to avoid ecological bias; this is achieved by centring the patient‐level covariate about its mean 88, 89, 90 (refer to Appendix section). If the within‐study and across‐study interaction effects are not separated in the model, then the resulting interaction effect is an amalgamation of the within‐study and the across‐study associations, which does not necessarily reflect the patient‐level interaction and may lead to misleading conclusions 89. Unfortunately, this issue is not well recognised, and in our experience, most researchers unknowingly fit the amalgamated interaction term in their one‐stage models.

The approaches are illustrated by the hypertension dataset again where the outcome is blood pressure and the summary parameter of interest is the patient‐level interaction of treatment effect with age. Riley *et al.* fitted one‐stage ANCOVA models that either amalgamated the interactions or separated them out 89. In the two‐stage approach, equation (1) was fitted with age and an interaction with age and treatment. A fixed interaction term was assumed for both one‐stage and two‐stage models, and results obtained using REML are shown in Table VII.

**Table VII:** One‐stage and two‐stage IPD meta‐analysis results for treatment–age interactions in the hypertension dataset according to within‐trial, across‐trial and amalgamated interactions.

Interaction covariate	One‐stage			Two‐stage
	Amalgamated interaction (95% CI), *p*‐value	Within‐trial interaction (95% CI), *p*‐value	Across‐trial interaction (95% CI), *p*‐value	Within‐trial interaction (95% CI), *p*‐value
Age	−0.067 (−0.094 to −0.040), <0.001	−0.050 (−0.116 to 0.017), 0.142	−0.071 (−0.100 to −0.041), <0.001	−0.049 (−0.115 to 0.017), 0.142

IPD, individual participant data; CI, confidence interval.

The one‐stage approach with the amalgamated interaction estimate suggests a statistically significant association between age and treatment effect (95% CI: −0.094 to −0.040). However, when the one‐stage analysis correctly separates the within‐trial and across‐trial interactions, the summary within‐trial interaction is no longer statistically significant (95% CI: −0.116 to 0.017), and gives results almost identical to the two‐stage approach. Therefore, the specification of within‐trial and across‐trial interactions in a one‐stage analysis can lead to differences with the two‐stage approach and can influence statistical and clinical conclusions.

## 4 Discussion

We have outlined the key framework for one‐stage and two‐stage IPD meta‐analysis models. Previous authors have compared one‐stage and two‐stage approaches through theoretical consideration 52, 53, 54, simulation 9, 49 and empirical examples 2, 9, 48, 49, and found that largely the two approaches produce similar results. Although we agree that this will generally be the case, Section 3 described 10 reasons why one‐stage and two‐stage results may differ to help the user to understand why differences may arise in their own IPD meta‐analyses and illustrated these using a range of examples, including both fixed‐effect and random effects models. Although in some examples the differences were small or qualitatively unimportant, in others, the clinical and/or statistical conclusions were affected.

Most differences between one‐stage and two‐stage approaches occur because of different modelling assumptions, including the specification of the likelihood and included parameters, the choice of fixed or random effects and the utilisation of correlation. Choosing a different estimation procedure may also lead to important differences, especially in the random effects setting. Another way of understanding this is that the different assumptions, parameter specifications and estimation methods lead to different percentage study weights in the one‐stage and a two‐stage meta‐analysis (Riley *et al. submitted*), and therefore, summary results can differ because of a change in weighting. However, when the same assumptions are made in both approaches (and these assumptions are plausible), and the same estimation method is used, the resulting estimates should be very similar, which was demonstrated by the hypertension data example in Section 2.4.

We note, quite importantly, that whilst the one‐stage versus two‐stage issue is mainly of concern in IPD meta‐analysis, one‐stage analyses are sometimes possible with published data, and the same similarities and differences apply. For example, for binary outcomes, the analyst can essentially reconstruct the IPD if they extract 2 × 2 contingency tables from each trial report and then proceed to perform one‐stage logistic regression analyses 91 (such as equation (16)), as long as no adjustment for covariates is required.

We focused on summary results and their 95% CIs throughout the article. However, we recognise that other measures may also be of interest such as 95% prediction intervals 92, for which differences may be even more pronounced. We also acknowledge that, although in our experience our 10 reasons are the most likely, the list is not exhaustive, and it may be possible to get differences for other reasons. For example, the meta‐analyst may include covariate adjustment for all studies in a one‐stage approach, but in the two‐stage approach may only include covariate adjustment for a subset of trials. The issue of missing data has also not been explicitly addressed in this paper. In particular, we did not consider differences in one‐stage and two‐stage results after the imputation of missing participant data, although recognising this is a growing area of interest 1, 93, 94, 95, 96, 97, 98. However, in our longitudinal example, we noted that missing outcome data is handled naturally in the one‐stage approach under a missing at random assumption, which allows more efficient results by accounting for correlation between time points. Another issue not previously mentioned is how automated selection procedures may lead to differences in one‐stage and two‐stage results, for example, in regard to identifying the best fitting (non‐linear) trend for a continuous predictor in a prognostic model 99. Abo‐Zaid gives an example where the one‐stage approach suggests a quadratic trend between age and log‐odds of death, whereas the two‐stage approach suggests that a linear trend is preferred in each study and so a pooled linear trend is then obtained 100. By considering the trends in each study separately, the two‐stage approach had lower power than the one‐stage approach to identify (at a pre‐defined significance level) more complex relationships. However, if a quadratic trend had been forced in each study and then combined, the pooled quadratic trend would have been very similar to that from the one‐stage analysis.

So, should researchers choose a one‐stage or a two‐stage approach? We do not believe there is a ‘blanket’ answer to this question as it depends on many factors, including the clinical question, the parameter(s) of interest, the desired specification of the model, the desired estimation method, the assumptions willing to be made, the potential for non‐convergence and missing data, and the likelihood of small study sizes and rare events. However, we make some specific recommendations in Box 1. In particular, we agree with Debray *et al.* that ‘when planning an IPD meta‐analysis, the choice and implementation of a one‐stage or two‐stage method should be pre‐specified in the protocol as occasionally they lead to different conclusions’ 3. Moreover, the exact specification of the models should also be pre‐specified where possible, for example, in regard to the choice of fixed or random effects, the handling of adjustment factors, how clustering within studies will be accounted for, whether ecological bias will be removed, and (for time‐to‐event outcomes) the specification of the baseline hazards. Once the analyst has considered the assumptions they want to make and decided that they are plausible in either a one‐stage or two‐stage framework, then they could adopt either method as they are likely to give very similar results. However, in some situations, the assumptions may be more plausible in the one‐stage approach. In particular, for situations of rare outcomes and/or small studies, the one‐stage approach is our preferred option, in order to use a more exact likelihood, and to avoid making assumptions about within‐study normality and known within‐study variances. However, even in this situation, the one‐stage approach may suffer from convergence issues, which are difficult to identify in advance. Furthermore, estimation options such as REML may only be available in a two‐stage approach, which may help reduce downward bias in estimates of between‐study variances in one‐stage ML models.

For such reasons, or if the best choice of model specification and/or estimation method are unclear, a sensible strategy is to pre‐specify that both one‐stage and two‐stage analyses will be undertaken, and their results compared with check whether conclusions are the same. If they differ, then the meta‐analyst should seek to understand why, and the 10 reasons outlined should help elicit the explanation.

**Box 1: Key recommendations for the adoption of one‐stage or two‐stage IPD meta‐analyses.**

- State prior to analysis whether a one‐stage or two‐stage approach will be used for an IPD meta‐analysis. If the best choice is unclear, or if estimation difficulties are a concern (e.g. due to rare outcomes), then it is sensible to state that both approaches will be undertaken, and results compared.
- Where both one‐stage and two‐stage approaches are undertaken, both should be reported in subsequent publications for transparency, and any important differences between them should be explained.
- The estimation method(s) should be specified in advance for both one‐stage and two‐stage approaches, as the choice can influence the findings and the magnitude of differences between the approaches.
- The model assumptions (e.g. likelihood specification, choice of fixed or random effects) and parameter specifications (e.g. handling of adjustment factors and baseline hazards in each trial) should be pre‐specified regardless of whether one‐stage or two‐stage approaches are used.
- For all outcome types where studies are expected to be small, and in particular, for binary and time‐to‐event outcomes that are rare (or extremely common), then a one‐stage approach is preferred, as it avoids the use of approximate normal sampling distributions, known within‐study variances, and continuity corrections that plague the two‐stage approach with an inverse variance weighting.
- Any one‐stage analysis should account for the clustering of participants with studies. This is best achieved by including a separate intercept (distinct baseline hazard) per trial. One could (if considered plausible) also assume trial intercepts are drawn from some distribution or, for time‐to‐event outcomes, assume baseline hazards are proportional across trials, but this will usually be unnecessary unless interest lies in the intercept or baseline hazard itself (e.g. for developing a prognostic model).
- In a one‐stage approach, it is best to include separate adjustment terms (when included) and separate residual variance terms (for continuous outcomes) for each trial, as this makes less assumptions than a model with common adjustment terms and residual variances. Only when estimation issues arise might it be necessary to move away from this, for which random effects on adjustment terms may be helpful.
- Where random effects models are used, consider methods to derive 95% CIs for the summary effect that account for full uncertainty in the estimated variances in the meta‐analysis. For example, for the two‐stage approach, the use of methods such as the HKSJ variance estimator and *t*‐distribution to estimate CIs might be considered, and for the one‐stage approach, the use of Kenward–Roger variance estimator and *t*‐distribution may be useful.
- A standard two‐stage approach does not automatically account for correlation between the parameters of the regression model estimated in the first stage; this may lead to loss in precision and different summary estimates than the one‐stage approach, which automatically accounts for such correlation. This is especially important if there are missing outcome data, for example, missing outcomes at some time points in a meta‐analysis of longitudinal data. To account for correlation in a two‐stage meta‐analysis, a multivariate model is required in the second stage.
- In a one‐stage IPD meta‐analysis, treatment–covariate interactions should be separated into within‐trial and across‐trial interactions to avoid ecological bias. This is automatically avoided in a two‐stage analysis when within‐study interaction estimates are obtained in each trial and then synthesised.

## Author contributions

RR and DB developed the research idea. DB undertook all the analyses under the supervision of RR and feedback from JE. DB drafted the paper and revised following comments from RR and JE.

## Appendix 1

### Treatment–covariate interactions in a one‐stage IPD meta‐analysis

A one‐stage approach must fit a model that separates the within‐study and across‐study interaction effects to avoid ecological bias. The one‐stage ANCOVA model (equation (14)) can be extended such that

$$Y_{ij}=\alpha_{j}+\beta_{i}y_{Bij}+\theta_{j}{\text{treat}}_{ij}+\beta_{j}z_{ij}+\gamma_{W}{\text{treat}}_{ij}*\left(z_{ij}-\bar{z_{j}}\right)+\varepsilon_{ij}$$

$$\theta_{j}=\alpha +\gamma_{A}\bar{z_{j}}+u_{j}$$

$$\varepsilon_{ij}\sim N\left(0,\sigma_{j}^{2}\right)$$

$$u_{j}\sim N\left(0,\tau^{2}\right)$$

Here, *z_ij_* denotes the covariate of interest, the fixed‐effect within‐study interaction effect is denoted by *γ_W_* and the across trials interaction is denoted by *γ_A_* (which is also the meta‐regression result). The unexplained between‐study variance of the treatment effect is given by *τ* ^2^, and the covariate is centred about the mean covariate value, $\bar{z_{i}}$ in each study to ensure that the within‐study interaction is separated from the across‐study interaction. If the within‐study and across‐study interaction effects are not separated in the model, then the resulting interaction effect is an amalgamation of the two different interaction effects 89.
